# Life as a new investigator

**DOI:** 10.1186/s40697-015-0083-4

**Published:** 2015-12-01

**Authors:** Darren Bridgewater

**Affiliations:** Department of Pathology and Molecular Medicine, McMaster University, Hamilton, Canada; Education Program in Anatomy, Hamilton Centre for Kidney Research, Pathology and Molecular Medicine, McMaster University Medical Centre, 1200 Main St. West, Hamilton, Ontario L8N 3Z5 Canada

## Life as a new investigator

I remember being an eager third year undergraduate student with frost-tipped hair, walking through the corridors of Children’s Hospital of Western Ontario with my supervisor Dr. Matsell, a pediatric nephrologist, to see one of his patients with congenital kidney disease. Dr. Matsell explained the patient’s kidneys had not developed properly, resulting in poor function and thus the need for dialysis. When I asked him why their kidneys failed to develop properly, he simply responded with a slight nod and frown that implied he did not know. At this moment, it clicked. I grasped the clinical impact of the kidney research I was conducting as a summer student. This defining moment has not only helped motivate me through my Ph.D. and post-doctoral fellowship but remains a touchstone for me as an independent investigator.

When I first started my laboratory, I was fortunate to share lab space with more established researchers and quickly learned to adopt the mantra of “beg, borrow, and steal” with an abundance of apologies and I.O.Us. In retrospect, integrating my lab with more established laboratories was fundamental to successful start up. These other researchers and their students provided invaluable technical support in experimental protocols, reagents, equipment, and knowledge of the inner workings of the university’s research core facilities. Another crucial early practice was hiring the right research personnel who had the ability to work independently with a high degree of self motivation, as the majority of my time was dedicated to developing my teaching curriculums, teaching, and writing grants.

The excitement of buying new equipment and reagents, hiring personal, maintaining transgenic mice, and performing experiments quickly turned to a sense of urgency as the invoices started rolling in and my start-up funds were quickly dwindling. Having had no luck in winning the lottery and realizing I had no choice but to pay these invoices, I jumped head first into the anxiety-provoking, stomach-churning process of writing grants. After my first grant was riddled with red marker by internal review, it was clear I had to develop my grant-writing skills. I therefore learned that identifying the right mentors and colleagues who cared about my success and would provide constructive feedback, no matter how painful, was the key to writing effective grants. With a newly found writing plan, experienced internal reviewers, and a smidgen of confidence, I applied for seven grants in my first year, and was awarded none. However, the reviewers’ comments from the granting agencies suggested a level of interest and enthusiasm for my research especially those from the Kidney Foundation of Canada. Therefore, with some positive reviews in hand, a little hope, guidance from mentors, and dedicated research staff, we addressed all the reviewers’ concerns, and in my second grant cycle, I finally received my first grant. Then second. Then third. Then fourth—most for modest amounts of money and for short duration. But at last, I had achieved grant success instrumental for career progression, and most importantly, this allowed me to further my research to generate foundational knowledge toward a cure for congenital kidney disease. I am indebted to the Hamilton Health Sciences New Investigator program, Bickell Foundation, NSERC, and Kidney Foundation of Canada for their support for me as a new investigator.

Sadly, the gratification of grant success is too short lived. With funding in hand and productive graduate students settling in, publishing became the next top priority. Again, the cycle of rejections and revisions reared its ugly head and led me to question my research abilities as a new investigator. However, my lab members’ persistence, combined with input from my colleagues, helped me realize this was part of the normal process for a new investigator in getting published. After two rejections and completing a “major revision,” I finally received a response from the editor. This was a milestone moment—I remember exactly where I was and what I was doing—vacillating whether or not to open the email or wait until after the Christmas holidays. Happily, a text arrived a few moments later from my student with the subject line “Let’s celebrate!” Suffice to say, our first manuscript as an independent laboratory had been accepted for publication, and the next day involved a headache and a mix of Tylenol and ibuprofen. We went through the same process for subsequent publications but with a notably reduced stress since I knew it was not just me and what I was doing wrong, but rather the normal process of manuscript peer review. Finally, after 3 years, I was able to lift my head out of the grant and publication fog and noticed my hair had turned gray. However, what I lost in hair color was recompensed by a new-found respect for seasoned researchers who are continually well-funded and well-published. I also realized this career was going to be a continuing battle and crazily enough, one that I would thoroughly enjoy.

In addition to research, my role at McMaster also includes a heavy teaching component. In fact, there is no doubt that my past teaching experiences during my Ph.D. and postdoc were instrumental in my receiving job offers and landing my academic position. In retrospect, my teaching experience was most likely my ticket into the system. Within weeks of setting up my laboratory, I started designing, implementing, and teaching the anatomy curriculum to first and second year medical students at the Michael G. DeGroote School of Medicine Regional campus in Waterloo. This was an exciting and unique opportunity because I was able to integrate my teaching philosophy into this new curriculum. Over the first few years, this required a significant time investment. But, the success of this program has led to several teaching awards and its implementation at our other campuses, thus making this one of the accomplishments I am most proud of as a new investigator.

This editorial would not be complete without recognizing the challenges I have faced as new investigator, including balancing colleagues’ expectations, performing numerous tasks for which I received no formal training, and most importantly achieving a work-life harmony. As the new kid on the block, the number of requests from colleagues to undertake academic responsibilities overwhelmed me. Just to name a few, these included grant review panels, graduate student committees, faculty search committees, curriculum design, the tenure and promotion committee, guest lectures, presentations, manuscript and grant reviews, presenting at research days and conferences, forming collaborations, etc. While all these seemed essential at the time, I was completely overwhelmed and very close to an early career meltdown that was affecting my research program, teaching, and family life. In addition, as a new investigator I found myself consistently performing tasks I was not familiar with. For example, I was exercising my accounting skills by budgeting my research program, I was a human resources manager when hiring and letting go of employees and addressing their concerns, and I was a negotiator when patiently communicating with administration and employees. Moreover, I was in perpetual catch 22, where I was feeling guilty by not spending time with my family, yet also guilty for not being at work. Once again, I relied on the advice of my more established colleagues. During this process, I learned some of the most valuable lessons that were paramount to maintaining my sanity. I learned to respectfully decline offers (i.e., I actually learned its okay to say “no”), I prioritized tasks that were going to be most beneficial to my career goals, and most importantly, I recognized the need to cultivate outlets outside of the academic bubble to relieve stress. On that note, I started spending more guilt-free time with my family and friends, coaching my daughter’s sports activities, having date nights with my wife, and spending “my time” training for triathlons. These changes in my attitude helped me become more productive at work, with less stress guilt, making the career much more enjoyable.

For me, the first 4.5 years as a new investigator have been riddled with incredible challenges that ultimately reaped incredible rewards. I have now completed the tenure and promotion processes, secured long-term CIHR funding, my laboratory is publishing regularly in the field of understanding kidney disease, and the teaching curriculums are established. But none of this would have been possible without relying on colleagues in the field with similar experiences and surrounding myself with individuals with similar career goals. And, the most important for me is to have outlets that you can always look to for motivation through the continuing challenges inherent to this career.

